# Supplementary material for: A partner-driven decision support model to inform the reintroduction of bull trout
Source: PLoS One. 2025 May 8;20(5):e0323427. doi: 10.1371/journal.pone.0323427 (PMC12061132; doi:10.1371/journal.pone.0323427)
Supplement: S1 File — (DOCX) [file pone.0323427.s001.docx]

Supporting information for **Decision support model for the feasibility of bull trout reintroductions.**

Joseph R. Benjamin^1^, Judith Neibauer^2^, Hugh Anthony^3^, Jose Vazquez^4^, Ashley Rawhouser^3^, and Jason B. Dunham^5^

^1^U.S. Geological Survey, Forest and Rangeland Ecosystem Science Center, Boise, ID, USA; [jbenjamin@usgs.gov](mailto:jbenjamin@usgs.gov); ORCID ID: 0000-0003-3733-6838

^2^U.S. Fish and Wildlife Service, (retired), currently residing in Peshastin, WA, USA; jneibauer9395@gmail.com

^3^National Park Service, North Cascades National Park, Sedro Woolley, WA, USA; [hugh_anthony@nps.gov](mailto:hugh_anthony@nps.gov), [ashley_rawhouser@nps.gov](mailto:ashley_rawhouser@nps.gov)

^4^U.S. Fish and Wildlife Service, Mid-Columbia Fish and Wildlife Conservation Office, Leavenworth, WA, USA; [jose_vazquez@fws.gov](mailto:jose_vazquez@fws.gov)

^5^U.S. Geological Survey, Forest and Rangeland Ecosystem Science Center, Corvallis, OR, USA; [jdunham@usgs.gov](mailto:jdunham@usgs.gov); ORCID ID: 0000-0002-6268-0633

S1 Table. Bull trout demographic model parameter abbreviations, descriptions and default values. Default values were modified form source data and agreed upon by participants.

| Abbreviation | Description | Value | Source |
| --- | --- | --- | --- |
| reintro.disc | Reintroduction discount | 0.8 | [1] |
| p.female | Proportion of females | 0.5 | assumed |
| f.Sp | Annual spawning probability of fluvial adults | 0.75 | [2] |
| r.Sp | Annual spawning probability of resident adults | 0.75 | [2] |
| a.Sp | Annual spawning probability of adfluvial adults | 0.75 | [2] |
| DI | maximum fry survival | 0.28 | [2] |
| G0 | Egg to fry survival | 0.4 | [2] |
| G3 | Resident subadult to adult survival | 0.15 | [2] |
| G5 | Fluvial subadult to adult survival | 0.1 | [2] |
| f.G2 | Juvenile to fluvial subadult survival | 0.07 | [2] |
| a.G2 | Juvenile to adfluvial subadult survival | 0.07 | [2] |
| f.egg | Eggs per fluvial adult female | 1400 | [3] |
| a.egg | Eggs per adfluvial adult female | 2430 | [3] |
| r.egg | Eggs per resident adult female | 625 | [3] |
| P2 | Survival and persistence as a juvenile | 0.03 | [2] |
| P3 | Survival and persistence as a resident subadult | 0.1 | [2] |
| P4 | Survival and persistence as a resident adult | 0.35 | [2] |
| P5 | Survival and persistence as a fluvial subadult | 0.15 | [2] |
| P6 | Survival and persistence as a fluvial adult | 0.55 | [2] |
| r.G2 | Juvenile to resident subadult survival | 0.15 | [2] |
| P7 | Survival and persistence as a adfluvial subadult | 0.15 | [2] |
| G7 | Adfluvial subadult to adult survival | 0.25 | [2] |
| P8 | Survival and persistence as a adfluvial adult | 0.55 | [2] |
| K | Juvenile carrying capacity per river kilometer | 500 | [4] |
| L | Lake Chelan predator discount | 0.8 | assumed |
| Yr.release | Consecutive years released | 5 | assumed |
| B | Brook trout discount (if present) | 0.9 | assumed |

1. Brignon WR, Peterson JT, Dunham JB, Schaller HA, Schreck CB. Evaluating trade-offs in bull trout reintroduction strategies using structured decision making. Canadian Journal of Fisheries and Aquatic Sciences. 2018 Feb;75(2):293–307.
2. Bowerman T. A multi-scale investigation of factors limiting bull trout viability. 2013 [cited 2024 Nov 5]; Available from: <https://digitalcommons.usu.edu/etd/1524>
3. Al- Chokhachy R, Budy P. Demographic characteristics, population structure, and vital rates of a fluvial population of Bull Trout in Oregon. Transactions of the American Fisheries Society. 2008;137(6):1709–22.
4. Benjamin JR, Brignon WR, Dunham JB. Decision analysis for the reintroduction of bull trout into the Lower Pend Oreille River, Washington. North American Journal of Fisheries Management. 2019 Oct;39(5):1026–45.

Any use of trade, firm, or product names is for descriptive purposes only and does not imply endorsement by the U.S. Government.
